# Relation of plasma *β*‐amyloid, clusterin, and tau with cerebral microbleeds: Framingham Heart Study

**DOI:** 10.1002/acn3.51066

**Published:** 2020-06-26

**Authors:** José Rafael Romero, Serkalem Demissie, Alexa Beiser, Jayandra J. Himali, Charles DeCarli, Daniel Levy, Sudha Seshadri

**Affiliations:** ^1^ Department of Neurology Boston University School of Medicine Boston Massachusetts; ^2^ NHLBI’s Framingham Heart Study Framingham Massachusetts; ^3^ Department of Biostatistics Boston University School of Public Health Boston Massachusetts; ^4^ Glenn Biggs Institute for Alzheimer’s and Neurodegenerative Diseases University of Texas Health Sciences Center San Antonio Texas; ^5^ Department of Neurology University of California‐ Davis Sacramento California; ^6^ The Population Sciences Branch of the National Heart, Lung, and Blood Institute of the National Institutes of Health Bethesda Maryland

## Abstract

**Objective:**

Cerebral microbleeds (CMBs) are associated with higher risk of stroke and dementia, predating clinical diagnosis by several years. CMB are considered markers of cerebral small vessel disease (CSVD): hypertensive (deep CMB) and cerebral amyloid angiopathy (lobar CMB). We related plasma *β*‐Amyloid (40, 42 and their ratio), clusterin, and tau levels to CMB to elucidate their role as biomarkers for the angiopathies represented by CMB.

**Methods:**

Dementia, stroke, and other neurological disease‐free Framingham Heart Study participants with available CMB and biomarker measurements were included. We related biomarker levels (standardized for analyses) to CMB presence overall and stratified by brain topography (any, lobar, deep), using multivariable logistic regression analyses.

**Results:**

CMB were observed in 208 (5.7%) participants (mean age 57 years, 54% women). After multivariable adjustment, A*β*1‐40 was associated with any CMB (OR (95%CI) 1.20 (0.99, 1.45) *P* = 0.062)) and lobar CMB (OR (95%CI) 1.33 (1.05, 1.68) *P* = 0.019), but not with deep CMB. Log‐A*β*1‐42 levels were not associated with CMB overall. Clusterin was related to mixed CMB (1.70 [1.05, 2.74], *P* = 0.031). Tau levels were associated with any CMB (OR (95%CI) 1.26 (1.07, 1.49) *P* = 0.006), lobar CMB (OR (95%CI) 1.26 (1.05, 1.52) *P* = 0.013), and with deep CMB (OR (95% CI) 1.46 (1.13, 1.89) *P* = 0.004).

**Interpretation:**

We found that plasma A*β*1‐40 and Tau are associated with CMB but further studies are needed to confirm their role in hemorrhage prone CSVD represented by CMB and as indicators of ongoing subclinical neuronal injury.

## Introduction

Cerebral small vessel disease (CSVD) is almost invariably present in mild degrees in persons with dementia,^1^ is estimated to account for about 30% of ischemic strokes,^2^ and constitutes the main cause of primary intracerebral hemorrhage.^3^ Cerebral microbleeds (CMBs) are subclinical radiological markers of hemorrhage‐prone CSVD, attributed to the main types of sporadic CSVD^4^: hypertensive vasculopathy (arteriolosclerosis) for deep CMB, and cerebral amyloid angiopathy (CAA) for lobar CMB. CMB are associated with higher risk of stroke and dementia,^5^ and may predate clinical diagnosis by several years, thus serving as markers to understand the pathophysiology of vascular contributions to neurodegeneration, dementia, and stroke risk in preclinical stages.

The use of plasma biomarkers has been advocated to characterize individuals in preclinical stages in the AD continuum.^6^ Similarly, there is much need for the identification of plasma biomarkers to characterize the types of CSVD most commonly seen in dementia and stroke. Plasma *β* amyloid (A*β*), clusterin, and tau are blood biomarkers that have been associated with dementia and stroke,^7^, ^8^, ^9^, ^10^ and may be involved in CSVD. A*β* plays a key role in the pathophysiology of AD and CAA,^11^ depositing primarily in brain parenchyma in the former and cerebral small vessels in the latter. A*β* 40 in particular has been reported to be deposited in cerebral vessels, while A*β* 42 is primarily deposited in brain parenchyma in persons with AD.^12^ Clusterin has been suggested to participate in clearance of A*β* via cerebral small vessels.^13^, ^14^ Higher clusterin plasma levels have been associated with severity of AD and rapid cognitive decline.^15^ The role of clusterin in AD, however, may depend on other factors such as A*β* levels, age, and APOE *ɛ*4 genotype.^10^ Tau plasma concentrations reflect ongoing neurodegeneration, have been associated with poor cognition and AD through mechanisms possibly independent from clusterin. CSF A*β* and tau levels have been related to CMB presence^16^ and higher clusterin levels have been associated with lobar CMB.^17^

Study of the relation of plasma clusterin, A*β*, and tau to CMB presence in a large sample of community dwelling individuals may provide insight into the pathophysiology of CAA and hypertensive vasculopathy in persons free of neurological disease. Study of the relation of these circulating biomarkers with CMB presence will allow further characterization of their role as biomarkers of CSVD represented by CMB topography, and to advance understanding of the role of CMB in neurodegeneration.

We hypothesized that higher clusterin, A*β*, and tau levels are associated with higher CMB presence; that these relations vary according to CMB topography; and explored whether these associations are modified by age, gender, and APOE *ɛ*4 genotype. We posit that the associations of clusterin levels with CMB will depend on A*β* levels, but be independent of plasma Tau levels.

## Methods

### Sample

The Framingham Heart Study began in 1948 with the enrollment of the Original cohort, followed in 1971 by enrollment of the Offspring cohort (children of the Original cohort and their spouses) and the Third Generation cohort (grandchildren of the Original Cohort participants and children of the Offspring cohort participants) in 2002. Participants from the three generations of Framingham Heart Study (FHS) Cohorts were included based on the availability of brain MRI allowing for CMB detection and biomarker data. Participants with prevalent stroke, dementia, or other neurological disease that could affect the estimation of CMB were excluded. Plasma biomarkers were obtained in FHS Original Cohort participants attending exam 23 (1992–1996, mean age 81 years) or 28 (2004–2005, mean age 89 years), Offspring Cohort participants attending exams 7 (1998–2001, mean age 62 years) or 8 (2005–2008, mean age 67 years), and Gen 3 Cohort participants attending exam 2 (2008–2011, mean age 47 years). As the number of participants with available plasma biomarker measurements was different for each biomarker, the samples differed between the biomarkers. A*β* was available only in Framingham Original and Offspring participants; among the participants who attended either of the respective exam cycle above, 1822 participants had brain MRI including CMB measurements. An additional 111 participants were excluded due to other neurological conditions affecting brain MRI. Clusterin was available only in Framingham Offspring (exam cycle 7) and Gen 3 (exam cycle 2) participants; among the participants who attended the respective exam cycle, 3832 participants had brain MRI including CMB measurements. An additional 170 participants were excluded due to other neurological conditions affecting brain MRI.

Tau measurements were available in the three Framingham Cohorts: at Original cohort exam cycle 28, Offspring cohort exam cycle 8, and at Third generation exam cycle 2; among the participants who attended the respective exam cycle, 3841 participants had brain MRI including CMB measurements. An additional 171 participants were excluded due to other neurological conditions affecting brain MRI.

Table 1 shows the sample selection chart for each biomarker. The Institutional Review Board of Boston University Medical Center approved the study protocol and informed consent was obtained from all subjects.

**Table 1:** Sample selection for each plasma biomarker.

Biomarker	*β* Amyloid			Clusterin			Tau		
Framingham Heart Study Cohort (Generation)	1	2	3	1	2	3	1	2	3
Exam cycle	23	7	–	–	7	2	28	8	2
Attended the exam and have biomarker data	772	3267	–	–	3290	3362	137	2885	3395
Exclusions									
No MRI →	730	1487	–	–	1500	1320	118	1115	1333
Sub‐total	42	1780	–	–	1790	2042	19	1770	2062
Other^1^ →	9	102	–	–	102	68	3	99	69
Total cohort available	33	1678	–	–	1688	1974	16	1671	1993
Final sample	1711			3622			3680		

^1^ Other refers to history of neurological conditions affecting brain MRI.

### Exposures

#### Plasma clusterin, A*β*, and Tau measurements

Details of blood sample collection, processing and quality control for measurement of A*β*, plasma clusterin and Tau have been previously reported.^18^ For A*β* the samples were analyzed at the Department of Molecular Pharmacology and Experimental Therapeutics of the Mayo Clinic, Jacksonville, FL. Quantification of A*β* in plasma was performed using INNO‐BIA assays (Innogenetics, Ghent, Belgium), which is a multiplex microsphere‐based Luminex xMAP technique. Intra‐assay coefficients of variations (CVs) for A*β*1–40 and A*β*1–42 were 3.2% and 2.6%, and interassay CVs were 10.5% and 7.6% respectively.^18^

Plasma clusterin levels were assessed as part of the Systems Approach to Biomarker Research (SABRe) project 2 using a Luminex xMAP assay.^19^ The Multiplex assay had five protein targets (Clusterin, Apo‐A1, Apo‐B100, Lp(a), and CRP) and used a plasma dilution of 1/10,000. The intra‐assay %CV for the clusterin measurements was 3.95%, and Interassay % CV was 8.4%. The assay had a lower limit of quantitation of 1.5 and upper limit of quantitation (ULOQ) of 2.67 × 10^4^, with a seven‐point calibration curve.^10^

Plasma tau was analyzed by Quanterix (Lexington) using a Simoa™ Tau 2.0 Kit and a Simoa HD‐1 analyzer. This assay is a molecule enzyme‐linked immunosorbent assay (digital ELISA) with a limit of detection of 0.019 pg/mL. The assay includes a set of monoclonal antibodies reacting to both normal and phosphorylated tau and can detect all tau isoforms. The analytical range was between 0.06 and 360 pg/mL. The intra‐ and interassay coefficients of variation were 4.1% and 7.5% respectively.^20^

### Outcomes

#### Brain MRI protocol

A 1.5‐tesla MR machine (Siemens Magnetom) was used to obtain the following sequences: coronal T2‐weighted 2470/20 to 80 (TR/ TE), echo train length 8, field of view 22 cm, acquisition matrix 192x256 interpolated to 256x256 with 1 excitation, 4‐mm slice thickness from nasion to occiput, sagittal T1‐weighted 11.4/4.4, 3D FLASH, 192 mm slab, 128 slices of 1.5‐mm thickness, 12‐degree flip angle and axial T2*gradient echo 656/26 (TR/TE), field of view 22 cm, acquisition matrix 144 × 256, 30‐degree flip angle, 19 slices of 5‐mm thickness, and 2 mm gap.

MRI were analyzed using a stroke visualization tool developed at the Imaging of Dementia and Aging (IDeA) laboratory that allows simultaneous visualization of multiple image sequences at three times magnification.

#### Cerebral microbleeds (CMB)

CMB presence, number, and topography were graded as per previously described methods using published guidelines.^4^ CMB topography was divided into lobar only (confined to cortex and subcortical white matter), deep only (internal capsule/external capsule, thalamus, basal ganglia, brainstem) or deep + mixed (deep only or concurrent lobar and deep location, including cerebellum). We also studied CMB burden within individuals graded as absent versus present (≥1 CMBs), ≥2 CMBs, or ≥5 CMBs, and the subgroup of 2 or more lobar only CMB representing probable CAA as per the recently modified Boston criteria. We have previously related the reproducibility of CMB ratings. The intrarater reliability based on blinded reading of 200 scans on two separate occasions was excellent (kappa statistic 0.78). Inter‐rater reliability comparing two independent readers in a subset of 200 scans was excellent (Kappa 0.78). Detailed description of our CMB rating methods have been published.^21^

### Clinical characteristics – covariates

Clinical and demographic characteristics were measured at examination cycle closest to the biomarker measurement. Hypertension was defined using JNC‐7 criteria as systolic blood pressure ≥140 mm Hg, diastolic blood pressure ≥90 mm Hg or use of antihypertensive medications. Current smoking was defined as self‐reported smoking of at least one cigarette per day within the year preceding examination. Medication use was ascertained by self‐report. Prevalent cardiovascular disease was defined as coronary heart disease, peripheral arterial disease and/or heart failure. APOE genotype was determined as previously described.^22^ We used presence of any ε4 allele, previously related to CMB presence and associated with biomarker measurements.^21^

### Statistical analyses

Descriptive statistics were obtained for the clinical, demographic, and biomarker variables (Tables 2, 3, 4). The distribution of A*β* was skewed, thus natural logarithmic transformation was used. Log A*β*, clusterin, and tau levels were standardized to 0 mean and unit standard deviation for analyses.

**Table 2:** Baseline characteristics of the study sample based on **Tau** sample, *N* = 3680.

	All *N* = 3680	CMB *N* = 208 (5.7%)	No CMB *N* = 3472 (94.3%)
Clinical characteristics (continuous), mean (SD)			
Age (years)	55 (13)	64 (13.6)	54.9 (13.2)
Time between biomarker measurement and MRI (years)	1.3 (1.2)	1.1 (1.2)	1.4 (1.3)
Clinical characteristics (categorical), *n* (%)			
Men	1708 (46.4%)	111 (53.4)	1597 (46)
Hypertension	1450 (39.4)	122 (58.7)	1328 (38.3)
Current smoking	314 (8.5)	10 (4.8)	304 (8.8)
Statin use	920 (28.2)	96 (49)	824 (26.9)
Antithrombotic use	800 (24.6)	81 (41.3)	719 (23.5)
Hypertension treatment	1199 (32.6)	104 (50.0)	1095 (31.6)
Plasma biomarkers, median (25th, 75th percentile)			
Tau pg/mL	3.9 (3.24, 4.69)	4.02 (3.3, 4.9)	3.9 (3.2, 4.7)
C‐reactive protein mg/L	1.31 (0.64, 2.92)	1.28 (0.7, 2.8)	1.31 (0.64, 2.92)
APOE genotype			
Any *ɛ*4 allele presence	800 (22.6)	44 (22.4)	756 (22.6)

**Table 3:** Baseline characteristics of the study sample based on ***β* Amyloid**, N = 1711.

	All *N* = 1711	CMB *N* = 142 (8.3%)	No CMB *N* = 1569 (91.7%)
Clinical characteristics (continuous), mean (SD)			
Age (years)	60 (9)	66 (8.9)	59.7 (9)
Time between biomarker measurement and MRI (years)	6.4 (2.1)	6.5 (2)	6.4 (2.2)
Clinical characteristics (categorical), *n* (%)			
Men	790 (46.2%)	80 (56.3)	710 (45.3)
Hypertension	655 (38.3)	74 (52.1)	581 (37)
Current smoking	189 (11.0)	14 (9.9)	175 (11.2)
Statin use	269 (15.7)	44 (31)	225 (14.3)
Antithrombotic use	23 (1.3)	6 (4.2)	17 (1.1)
Hypertension treatment	466 (27.2)	56 (39.4)	410 (26.1)
Plasma biomarkers, median (25th, 75th percentile)			
*β* Amyloid 40 pg/ml	153.5 (137.3, 174.4)	163.2 (144.4, 183.8)	152.7 (136.6, 173.5)
*β* Amyloid 42 pg/ml	42.9 (37, 49)	42.8 (36.7, 48.8)	42.9 (37.1, 49.0)
*β* Amyloid 42/40 ratio	0.28 (0.24, 0.31)	0.26 (0.22, 0.29)	0.28 (0.24, 0.31)
C‐reactive protein mg/L	1.9 (0.9, 4.5)	2.4 (1.1, 5.1)	1.82 (0.88, 4.43)
APOE genotype			
Any *ɛ*4 allele presence	378 (22.6)	32 (23.2)	346 (22.5)

**Table 4:** Baseline characteristics of the study sample based on **Clusterin**, *N* = 3662.

	All *N* = 3662	CMB *N* = 212 (5.8%)	No CMB *N* = 3450 (94.2%)
Clinical characteristics (continuous), mean (SD)			
Age (years)	52 (11)	60 (11.3)	52 (11)
Time between biomarker measurement and MRI (years)	3.8 (2.8)	4.7 (2.9)	3.8 (2.8)
Clinical characteristics (categorical), *n* (%)			
Men	1712 (46.8%)	114 (53.8)	1598 (46.3)
Hypertension	1090 (29.8)	94 (44.3)	996 (28.9)
Current smoking	362 (9.9)	16 (7.5)	346 (10.0)
Statin use	542 (16.7)	64 (32)	478 (15.7)
Antithrombotic use	109 (3.4)	10 (5)	99 (3.3)
Hypertension treatment	806 (22.0)	72 (34.0)	734 (21.3)
Plasma biomarkers, median (25th, 75th percentile)			
Clusterin μg/mL	51.4 (44.0, 63.8)	51.3 (44.0, 64.3)	51.4 (43.9, 59.4)
C‐reactive protein mg/L	1.5 (0.7, 3.6)	1.5 (0.7, 3.5)	1.9 (0.8, 4.5)
APOE genotype			
Any *ɛ*4 allele presence	802 (22.7)	46 (22.9)	756 (22.7)

We used multivariable logistic regression analyses to estimate odds ratios (OR) and 95% confidence intervals for the association of plasma clusterin, A*β*, and Tau levels with CMB outcomes (any CMBs, lobar, deep, mixed location). We first assessed if each of the biomarkers was associated with CMB presence in models adjusted for age, gender, cohort‐generation, and time between biomarker and CMB measurements. We then evaluated a multivariable adjusted model including covariates that are known to be associated with CMB presence or considered potential confounders: hypertension, statin use, antithrombotic use, and APOE *ɛ*4 status. A third model was additionally adjusted for C‐reactive protein levels because prior studies suggest that clusterin levels may be related to inflammation,^23^ (Table 3). A fourth model was additionally adjusted for white matter hyperintensity volume and covert brain infarcts to assess if the associations were independent of ischemic markers of small vessel disease.

For each biomarker we conducted stratified analysis by the following factors: age (55 years old or older vs. younger), gender, and APOE genotype (any *ɛ*4 risk allele vs. none). For clusterin, we additionally conducted stratified analyses by plasma A*β* levels (above vs. below median). We evaluated nonlinear associations by relating tertiles of each biomarker (with the lowest tertile as referent) to CMB presence. Lastly, we performed separate exploratory logistic regression analyses to assess the association of biomarker levels and CMB burden, with three CMB burden groups: ≥1, ≥2, and ≥5 CMB, with no CMB as reference group; and with the subgroup of 2 or more lobar only CMB representing probable CAA. All analyses were performed using SAS version 9.4 (Cary, NC). A *P*‐value <0.05 was considered statistically significant.

## Results

We observed CMB in 208 (5.7%) participants, with a predominant lobar location: 73% lobar only versus 16.3% deep only and 10.6% mixed. The mean (SD) age in our participants was 55 (13) and 54% were women. Participants with CMB were older, had a higher prevalence of hypertension, were more likely to use statin and antithrombotic treatments (Tables 2, 3, 4).

### Primary results (model 2, Table 5)

After adjusting for vascular risk factors and APOE *ɛ*4 genotype, we observed a borderline association between A*β*1‐40 with any CMB presence (OR [95% CI] 1.20 [0.99, 1.45], *P* = 0.06) and a stronger association with lobar CMB (OR [95% CI] 1.33 [1.05, 1.68], *P* = 0.019). There was a borderline association with mixed only CMB, attenuated after adjustment for vascular risk factors. No significant association was observed between A*β*1‐40 and deep only or deep and mixed CMB, or A*β*1‐42 and any of the CMB groups. The A*β*1‐42/1‐40 ratio was significantly associated with any CMB and borderline associated with lobar CMB group, suggesting that the relationship with CMBs and the A*β*1‐42/1‐40 ratio was likely driven by A*β*1‐40 (Table 5).

**Table 5:** Logistic regression analyses for the association of biomarkers and prevalent CMB.

Biomarker	Model	Prevalent CMB by brain topography				
		All CMB (*n* = 142/212/208)^1^	Lobar only (*n* = 95/152/152)^1^	Deep only (*n* = 26/37/34)^1^	Any Deep [deep + mixed] (*n* = 47/60/56)^1^	Mixed only (*n* = 21/23/22)^1^
		OR (95% CI), *P*‐value	OR 95% CI *P*‐value	OR 95% CI *P*‐value	OR 95% CI *P*‐value	OR 95% CI *P*‐value
A*β* 40 (*N* = 1711)^2^	1	1.21 (1.00, 1.45) 0.048	1.30 (1.03, 1.63) 0.027	0.84 (0.60, 1.14) 0.248	1.07 (0.80, 1.43) 0.670	1.57 (0.98, 2.51) 0.062
	2	1.20 (0.99, 1.45) 0.062	1.33 (1.05, 1.68) 0.019	0.83 (0.60, 1.14) 0.248	1.02 (0.76, 1.36) 0.899	1.42 (0.89, 2.25) 0.137
A*β* 42 (*N* = 1711)^2^	1	0.96 (0.81, 1.14) 0.656	1.02 (0.82, 1.26) 0.865	0.74 (0.52, 1.07) 0.108	0.86 (0.65, 1.14) 0.292	1.04 (0.68, 1.59) 0.873
	2	0.96 (0.80, 1.15) 0.641	1.03 (0.83, 1.29) 0.765	0.75 (0.52, 1.07) 0.116	0.84 (0.63, 1.11) 0.226	0.99 (0.65, 1.49) 0.944
A*β* 42/40 ratio (*N* = 1711)	1	0.81 (0.67, 0.97) 0.021	0.81 (0.65, 1.01) 0.058	0.89 (0.60, 1.33) 0.575	0.80 (0.58, 1.08) 0.144	0.69 (0.43, 1.09) 0.109
	2	0.81 (0.67, 0.97) 0.025	0.80 (0.64, 1.01) 0.059	0.91 (0.61, 1.35) 0.645	0.81 (0.59, 1.10) 0.176	0.70 (0.44, 1.11) 0.130
^3^Clusterin (*N* = 3662)	1	0.99 (0.84, 1.17) 0.931	1.02 (0.85, 1.23) 0.815	0.57 (0.35, 0.94) 0.028	0.90 (0.64, 1.27) 0.785	1.62 (1.04, 2.53) 0.034
	2	0.98 (0.82, 1.17) 0.798	0.99 (0.80, 1.21) 0.901	0.55 (0.31, 0.98) 0.042	0.95 (0.66, 1.38) 0.785	1.70 (1.05, 2.74) 0.031
^3^Tau (*N* = 3680)	1	1.07 (0.99, 1.16) 0.093	1.07 (0.98, 1.17) 0.142	1.09 (0.98, 1.21) 0.117	1.07 (0.95, 1.21) 0.284	0.62 (0.28, 1.40) 0.252
	2	1.26 (1.07, 1.49) 0.006	1.26 (1.05, 1.52) 0.013	1.46 (1.13, 1.89) 0.004	1.23 (0.90, 1.69) 0.191	0.55 (0.24, 1.25) 0.153

Abbreviations: CI, confidence interval; CMB, cerebral microbleeds; OR, odds ratio. A*β* = beta amyloid.

Model 1 is adjusted for age, gender, and time between exam and MRI. Model 2 is additionally adjusted for hypertension, statin use, antithrombotic use, and APOE *ɛ*4 genotype.

^1^ Number of participants with CMB according to biomarker (A*β* **,** Clusterin**,** Tau)

^2^ Log‐transformed and standardized to 0 mean and unit standard deviation.

^3^ Standardized to 0 mean and unit standard deviation.

The relation of clusterin to CMB presence seemed to vary depending on the topography of CMB. We did not observe any significant associations of clusterin levels with presence of any CMB or lobar CMB. Although an inverse association was observed with presence of deep only CMB in model 1 (OR [95% CI] 0.55 [0.31, 0.96], *P* = 0.037), further multivariable adjusted models were unstable due to the small sample of participants with CMB in this subgroup. Higher clusterin levels were associated with mixed only CMB (OR [95% CI] 1.70 [1.05, 2.74], *P* = 0.031).

Tau plasma levels were associated with any CMB presence (OR [95% CI] 1.26 [1.07, 1.49], *P* = 0.006), with strictly lobar CMB (OR [95% CI] 1.26 [1.05, 1.52], *P* = 0.013), and deep only CMB presence (OR [95% CI] 1.46 [1.13, 1.89], *P* = 0.004). Tau levels were not associated with mixed CMB. Further exploratory analyses to assess for additional confounding on this result showed that the proportion of participants using cardiovascular disease prevention was highest among participants with mixed CMB (statin use 81%, antithrombotic therapy 61.9%, antihypertensive treatment 72.7%).

Additional adjustment for CRP levels in model 3 did not change the association between any of the biomarkers and CMB presence (data not shown). Similarly, additional adjustment for MRI markers of ischemic small vessel disease, white matter hyperintensity volume and presence of covert brain infarcts, did not change the results. Overall the associations were linear, with no clear threshold observed when comparing quartiles of the biomarkers.

The prespecified stratified analyses (i.e., by age, gender, and APOE *ɛ*4 status for clusterin, A*β*, and Tau, and by A*β* levels for clusterin) did not reveal any meaningful differences in the association of the biomarkers with CMB presence. Analyses of the relation between biomarker levels and CMB burden were limited by the small sample of participants with high CMB burden, but showed that the strength of association between CMB and A*β*1‐40 and the A*β*1‐42/1‐40 ratio increased as CMB burden increased. For A*β* 1‐40 the respective associations with CMB count groups (1, ≥2, ≥5) were OR [95% CI] 1.19 (0.95, 1.48), *P* = 0.127; 1.24 (0.88, 1.74), *P* = 0.226; 1.90 (1.04, 3.45), *P* = 0.036. For the A*β*1‐42/1‐40 ratio the OR [95% CI] were 0.84 (0.68, 1.04), *P* = 0.115; 0.72 (0.51, 1.02), *P* = 0.066; 0.63 (0.37, 1.08), *P* = 0.096 respectively.

Analyses in the subgroup of probable CAA (i.e., 2 or more lobar only CMB) were limited by the small number of participants, but significant in the relation with Tau (OR [95%CI] 1.39 [1.03, 1.87], *P* = 0.030).

## Discussion

Our study included a large sample of community dwelling middle aged individuals that were free of neurological disease at the time of biomarker measurement and brain MRI. We observed that plasma A*β*1‐40, the A*β*1‐40/1‐42 ratio, and tau levels were associated with CMB presence overall and with strictly lobar CMB in particular. Clusterin was associated with higher odds of mixed location CMB, and lower odds of strictly deep CMB but multivariate analyses were limited by small number of CMB in the latter subgroup. There was an association between plasma tau and deep CMB, while there were no significant associations with mixed CMB.

### A*β* and CMB

In agreement with prior studies including smaller samples of elderly persons and individuals with impaired cognition and dementia,^12^ we observed that A*β*40 but not A*β*1‐42 was associated with CMBs and the A*β*42/40 ratio showed an inverse relation with markers of SVD including CMB. However, the same study also reported an association of A*β*40 with lobar and deep CMB, in contrast with our findings of a significant association only with lobar CMB. This difference may be due to differences in the samples studied, such as older age and higher prevalence of hypertension in their study, i.e., a sample more likely to have more advanced forms of both types of angiopathy represented by lobar and deep CMB. In another study in elderly and patients with impaired cognition (Swedish BioFINDER study Biomarkers for Identifying Neurodegenerative Disorders Early and Reliably) it was found that higher plasma A*β*40 and A*β*42 levels were associated with CMB presence.^8^ The differential findings of an association of A*β*1‐40 but not A*β*42 with CMB in our study, in particular lobar CMB, is supportive of the vascular deposition of the more soluble A*β* 40, whereas A*β* 42 is a predominant component in amyloid plaques in AD. However, it is possible that A*β* 42 also becomes related to CMB in more advanced stages of angiopathy or neurodegenerative process as can be seen in older age and in impaired cognition as in the Swedish BioFINDER study sample. This is also suggested by the observation that the associations of A*β*40 with mixed only CMB were stronger in our study, though not statistically significant likely due to the small sample of participants with mixed CMB.

Our finding of low A*β*42/A*β*40 ratio associations with CMB is in line with prior reports showing that decreased ratios are associated with higher A*β* brain burden.^24^ Given that lobar CMB are considered to represent predominantly CAA (in support FHS participants with lobar CMB have higher prevalence of APOE *ɛ*4 genotype), our findings would suggest that plasma A*β*1‐40 may play a role in hemorrhage prone CSVD in preclinical stages, but should be confirmed in additional studies.

Of note, plasma A*β* may not be a marker solely for hemorrhagic CSVD as prior reports have shown that higher A*β*40 levels may also be increased in participants with lacunar infarctions and WMH,^12^ and in elderly with CAA.^25^

### Clusterin and CMB

Clusterin levels have been related to dementia and stroke, but the relation is complex and influenced by several factors. Although we observed an inverse association of higher clusterin levels with lower odds of CMB in deep brain regions in our initial model, multivariate adjustment was not possible. Our results do not support a role for clusterin in the vascular injury represented by CMB in lobar regions; with regards to a potential role in deep CMB, presumably increased clusterin levels may reflect an attempt to prevent ongoing neurodegeneration that may be sufficient to prevent deep CMB representing hypertensive vasculopathy; however, as higher CMB burden is accrued, clusterin may be ineffective to prevent CMB as suggested by the observation that higher clusterin levels are associated with higher odds of mixed only CMB. However, these results should be interpreted with caution and taken only as hypothesis generating, in view of the small sample of persons with deep only and mixed CMB.

### Tau and CMB

The association we observed of higher tau levels with higher CMB presence overall, and lobar and deep in particular, signal that CMB may also be markers of ongoing neurodegeneration. The association of tau levels with mixed only CMB, a subgroup representing higher burden of CMB, though not statistically significant was surprisingly in the opposite direction than with deep CMB. To further understand this finding, we assessed for possible imbalance in covariates that would suggest residual confounding. We observed substantial imbalance in the proportion of participants taking treatments for cardiovascular disease prevention in the mixed CMB subgroup (more likely to use) compared with those with deep only CMB (less likely to use), suggesting that treatment effects are possibly implicated in these findings. Our multivariable analyses considered these treatments as covariates and adjusted analyses were large unchanged; in addition, we assessed for interaction with treatment use, but interactions were not significant. However, treatment related effects such as duration of exposure to medications, control achieved over the long‐term could not be accounted for in our analyses, and may represent residual confounding. It is also important to highlight that these analyses are limited by the small sample of mixed CMB outcomes.

Our findings support a role for tau in the group of probable CAA (i.e., 2 or more CMB). Given that the neurovascular unit (neuron‐blood vessel) is a fundamental component of the brain where neuronal structure and function are closely inter‐related to blood vessels, our findings suggest that vascular disease represented by CMB also indicates neuronal injury, but the relation may be modulated by cardiovascular treatments.

### Strengths and Limitations

Among the strengths in our study are the inclusion of a large sample with available biomarker and MRI data, high reliability of exposure and outcome ascertainments, both blinded to each other and to all clinical and demographic characteristics, and use of highly sensitive assays for biomarker measurements.^26^ Our study expands previous studies by including a large sample of middle aged community dwelling individuals.

Several limitations are noteworthy. Participants who underwent brain MRI are generally healthier than those who did not have MRI; however, the effect estimates are expected to be unbiased with respect to participant selection as inclusion into the present study is considered unrelated to the exposure or outcome. Our analyses involving prespecified subgroups of CMB burden are limited by the smaller sample among high burden subgroups. Analyses of the relation of biomarkers with mixed CMB are also limited by the small sample of outcomes in this group, precluding further exploratory analyses. The relation of the plasma biomarkers and CMB ratings is cross sectional, thus we cannot establish causality in their relationship. Our results, particularly in the relation of A*β* and CMB, should be considered hypothesis generating and interpreted with caution given the multiple comparisons performed. The generalizability of our results is limited to participants of White racial group, as Framingham Heart Study participants are primarily of European ancestry; this observation is important as the risk factors for CMB and underlying vasculopathy may differ in other racial groups. Similarly, given the complexity of our samples further studies are needed to replicate our results, particularly in samples of other racial and ethnic groups.

## Conclusion

We found plasma A*β* and Tau are related to CMB overall and lobar CMB in particular. Our results advance our understanding of the pathophysiology of CMB and their role in neurodegeneration. Our findings provide important insight into the mechanism of vascular disease predating the onset of dementia and stroke, and generate hypotheses to be tested in additional studies, for the possible role of these biomarkers in hemorrhage prone CSVD.

## Author Contributions

Study concept and design: JRR, SD, AB, SS; Acquisition, analysis, or interpretation of data: All authors. Drafting of the manuscript: JRR, SD. Critical manuscript revision: All authors.

## Conflict of Interest

Nothing to report.
